# MFAP5 inhibits the malignant progression of endometrial cancer cells *in vitro*

**DOI:** 10.1515/biol-2022-0990

**Published:** 2024-12-31

**Authors:** Guanying Liang, Zijuan Qi, Chun Du

**Affiliations:** Department of Pathology, Harbin Medical University Cancer Hospital, No. 150, Haping Road, Harbin, Heilongjiang, 150081, China; Department of Pathology, Heilongjiang Provincial Hospital, Harbin, Heilongjiang, 150000, China

**Keywords:** endometrial cancer, MFAP5, proliferation, EMT, AKT/mTOR

## Abstract

To investigate the biological role of MFAP5 in endometrial cancer (EC). HEC-1-A and Ishikawa cells overexpressing MFAP5 were created. Cell proliferation, apoptosis, migration, and invasion were evaluated using CCK8, colony formation, flow cytometry, and transwell assays. A western blot was used to analyze the expression of markers affiliated with the epithelial–mesenchymal transition process and AKT/mTOR pathway. As a result, MFAP5 was found to be down-regulated in EC. Overexpression of MFAP5 suppressed proliferation and promoted apoptosis of HEC-1-A and Ishikawa cells, as evidenced by the inhibition of cell viability and colony formation, and the increase in cell apoptosis rate. Besides, overexpression of MFAP5 attenuated the abilities of cell migration and invasion, as well as reduced MMP2 and MMP9 protein expression. Furthermore, E-cadherin protein level was elevated, while N-cadherin and α-SMA protein levels were decreased, and the phosphorylation of AKT and mTOR was reduced in cells overexpressing MFAP5. Our findings indicate that MFAP5 overexpression inhibits the malignant behaviors of EC cells, possibly by blocking the AKT/mTOR pathway, suggesting that MFAP5 may be a new therapeutic target for EC.

## 1 Introduction

Endometrial cancer (EC) is a gynecological tumor occurring in the endometrium [1]. In China, the incidence of EC and its mortality are on the rise every year, posing a serious threat to society [2]. Several factors, including tumor stage, histological types, and age, determine the prognosis of EC [3,4]. Based on statistics, the 5-year survival rate of EC patients that are diagnosed and treated early exhibits a range of 70–90%, whereas those diagnosed late typically experience 20–60% due to tumor metastasis [2,5]. Recent advancements in gene-targeted therapy for EC have expanded treatment options, offering patients more precise and efficacious interventions, with the potential to enhance future survival rates and quality of life [6–8].

Microfibril-associated protein 5 (MFAP5), an important extracellular matrix (ECM) protein, regulates the structure and function of ECM by mainly binding to microfibrils [9]. In cancer, MFAP5 was found to function in tumor growth, metastasis, and angiogenesis [10]. For example, MFAP5 may activate the epithelial–mesenchymal transition (EMT) program to promote basal-like breast cancer growth and aggressiveness [11]. MFAP5 modulated EMT-related pathways, which led to a reduction in cervical cancer cell migration and invasion [12]. MFAP5 facilitated the proliferation and metastasis of breast cancer cell, as reflected by the elevated levels of matrix metalloproteinase 2 (MMP2) and MMP9 after MFAP5 overexpression [13]. However, there is no clear understanding of MFAP5’s role in EC.

Herein, down-regulated MFAP5 was found by retrieving the TCGA database (https://*tcga*-data.nci.nih.gov/*tcga* ). Subsequently, a series of *in vitro* experiments was conducted to investigate the effect of MFAP5 expression on the malignant behavior of EC cells.

## 2 Materials and methods

### 2.1 Cell culture

Normal human cervical epithelial cells (HcerEpic) and EC cell lines (JEC, KLE, HEC-1A, and Ishikawa) were purchased from ATCC (Rockville, MD, USA). Cells were grown in DMEM medium with 10% FBS at 37°C with 5% CO_2_.

### 2.2 Cell transfection

Adenovirus vector overexpressing MFAP5 (ad-MFAP5) and adenovirus-negative control (ad-NC) were prepared by Genepharma (Shanghai, China). Then, transfection of ad-NC or ad-MFAP5 was conducted with 50 multiplicity of infection for 48 h. The blank group (Control) was taken as a control for the experiment.

### 2.3 CCK8 assay

Cells (1,000 cell/well) were planted into 96-well plate for 48 h. Whereafter, each well was injected with 10 µL of CCK8 reagent (Dojindo, Shanghai, China). A 2 h incubation was followed by the measurement of absorbance at OD 450 nm and the determination of cell viability.

### 2.4 Colony formation assay

Cells (2,000 cell/well) were inoculated into six-well plates and grown at 37°C for about 2 weeks. Afterward, a 30 min fixation with methanol was followed by staining the cells with 0.2% crystal violet. Photographs of the colonies were captured with a camera.

### 2.5 Flow cytometry

After collecting the cells, they were washed with buffer containing PBS. Following that, according to Annexin V-FITC detection kit (Dojindo), cells were stained with FITC-conjugated Annexin V and PI. A flow cytometry (FC500, BECKMAN COULTER, USA) was employed to analyze cell apoptosis within 1 h.

### 2.6 Transwell assays

Cells were suspended in serum-free medium and added to the upper compartment of a transwell. The lower compartment contained medium with 10% FBS. After cultivating for 24 h at 37°C, the upper chamber was removed, fixed in methanol, and stained with Giemsa. After cleaning, unmigrated cells were removed using a cotton swab. Next, the cells were visualized under the microscope (Leica Microsystems, Wetzlar, Germany) and counted. For cell invasion assay, a layer of Matrigel matrix was applied to the bottom of the transwell chamber.

### 2.7 Western blot

Cell lysates were prepared using RIPA buffer, then centrifuged and denaturated. Protein samples were then run on a 10% SDS-PAGE and transferred to PVDF membranes. After blocking, incubation of primary antibodies on the membranes at 4°C overnight was followed by secondary antibody incubation for 1 h. Table 1 lists the primary antibodies used. An ECL luminescent solution was utilized to render the color, and the protein bands were analyzed and quantified using Image J.

**Table 1:** Antibodies used for western blot

Antigen	Code	Working dilution	Supplier
MFAP5	ab171737	1:1,000	Abcam
MMP2	ab235167	1:1,000	Abcam
MMP9	ab137867	1:1,000	Abcam
E-cadherin	ab238099	1:1,000	Abcam
N-cadherin	ab207608	1:1,000	Abcam
α-SMA	ab108424	1:1,000	Abcam
p-mTOR	ab131538	1:500	Abcam
mTOR	ab134903	1:10,000	Abcam
p-AKT	ab314038	1:2,000	Abcam
AKT	ab179463	1:10,000	Abcam
GAPDH	ab59164	1:2,000	Abcam

### 2.8 Statistical analysis

Each experiment was conducted three times in triplicate. Data were analyzed using GraphPad Prism 8.0, presented as mean ± SD, and compared among groups using one-way ANOVA with a significance level of *p* < 0.05.

## 3 Results

### 3.1 MFAP5 is down-regulated in EC

**Figure 1:**
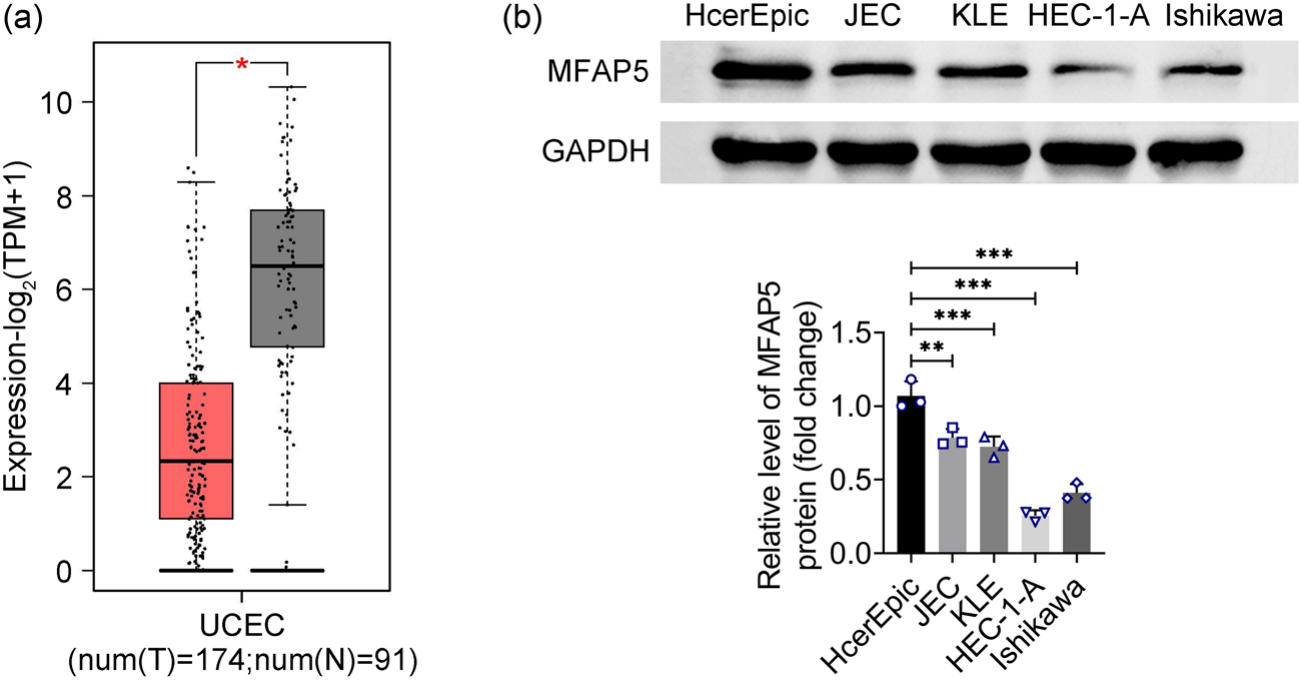
MFAP5 is down-regulated in EC. (a) Analysis of MFAP5 expression in 174 UCEC tumors on TCGA. (b) MFAP5 protein expression in cells was analyzed with the western blot method. ^*^ *p* < 0.05, ^**^ *p* < 0.01, and ^***^ *p* < 0.001.

Based on TCGA analysis, the results revealed that MFAP5 expression was lower in EC tissues compared to normal tissues (Figure 1a). Subsequent results from western blot showed that MFAP5 protein expression was observably decreased in EC cell lines (Figure 1b). These data suggest that MFAP5 is expressed at low levels in EC.

### 3.2 MFAP5 overexpression inhibits proliferation and promotes apoptosis of EC cells

**Figure 2:**
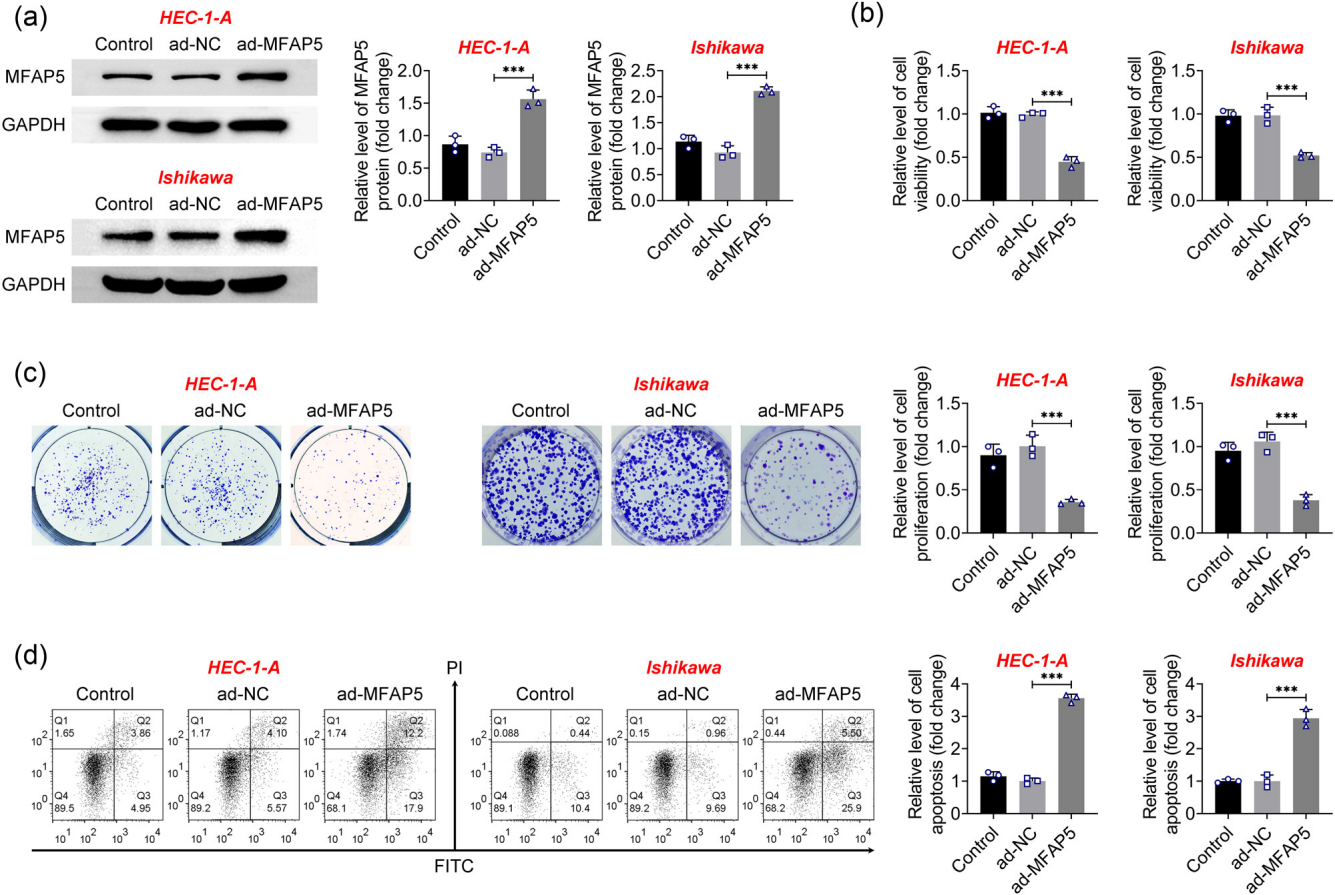
MFAP5 overexpression inhibits proliferation and promotes apoptosis of EC cells. (a) Overexpression efficiency of MFAP5 was detected by western blot. (b) Cell viability was evaluated using the CCK8 assay. (c) Cell clonogenic ability was examined by colony formation assay. (d) Apoptosis of cells was analyzed by flow cytometry. ^***^ *p* < 0.001.

An overexpression of MFAP5 in HEC-1-A and Ishikawa cells was conducted to study the function of the protein in EC. Western blot results showed a significantly elevated protein level of MFAP5 in cells after MFAP5 overexpression (Figure 2a). Furthermore, CCK8 assay and colony formation assay indicated significantly reduced cell viability and clonogenic activity in cells overexpressing MFAP5 compared to the control cells (Figure 2b and c). Moreover, results of flow cytometry showed that the MFAP5 overexpression group displayed a significant increase in apoptosis rates (Figure 2d). These data demonstrate that overexpression of MFAP5 suppressed proliferation and facilitated apoptosis of EC cells.

### 3.3 MFAP5 overexpression attenuates migration and invasion of EC cells

**Figure 3:**
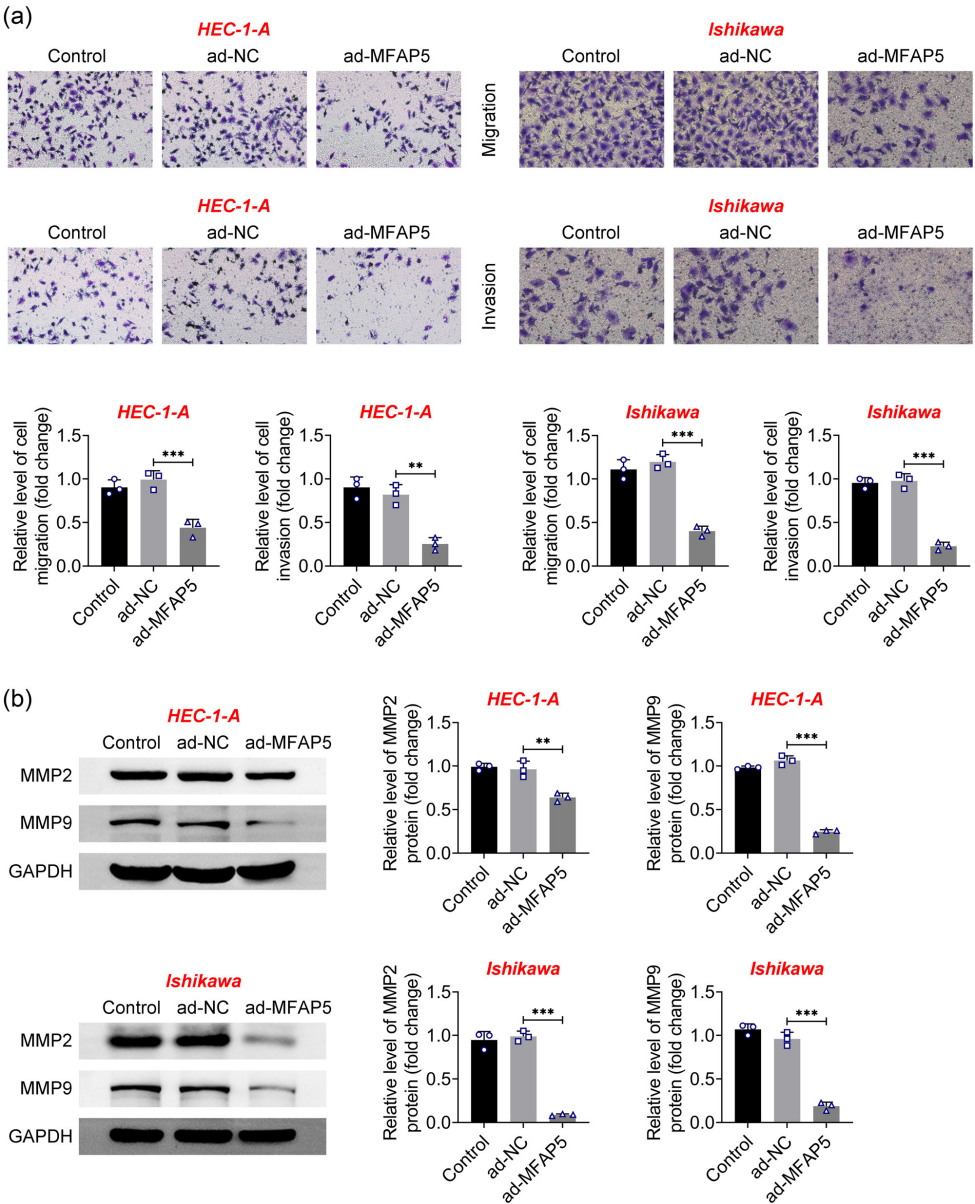
MFAP5 overexpression attenuates migration and invasion of EC cells. (a) Cell migration and invasion were assessed by transwell assays. (b) Western blot was used to detect MMP2 and MMP9 protein expression. ^**^ *p* < 0.01 and ^***^ *p* < 0.001.

Further, MFAP5 was examined for its impact on cell migration and invasion by transwell assays. As shown in Figure 3a, comparing the overexpressing MFAP5 group with the control and ad-NC groups, the number of migrating and invading cells was both significantly reduced. In addition, western blot results showed that MMP2 and MMP9 protein levels were observably decreased in the MFAP5 overexpression group (Figure 3b). These data indicate that overexpression of MFAP5 in EC cells inhibited their migration and invasion capabilities.

### 3.4 MFAP5 overexpression suppresses EMT in EC cells

**Figure 4:**
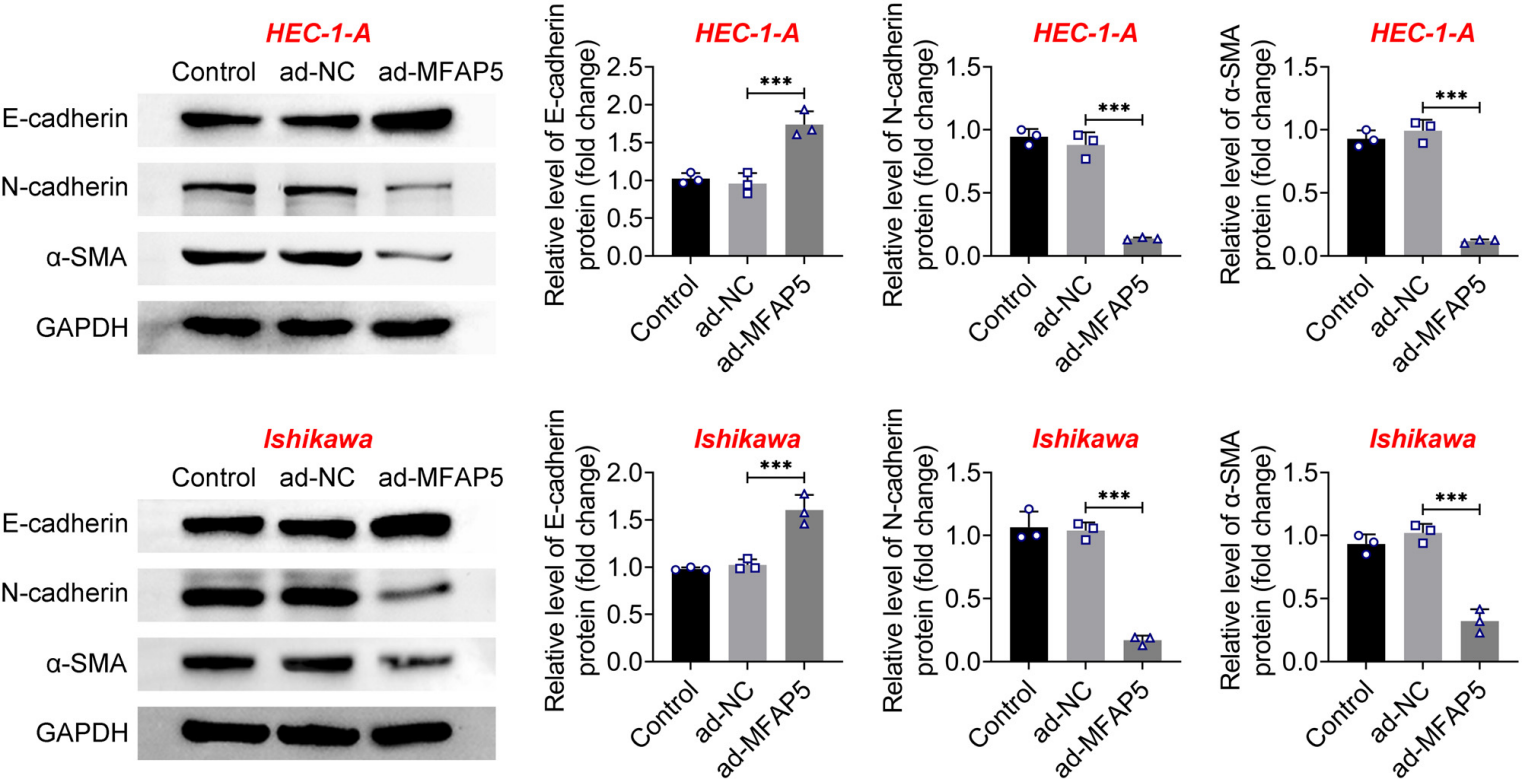
MFAP5 overexpression suppresses EMT in EC cells. E-cadherin, N-cadherin, and α-SMA protein expression in cells was tested through western blot assays. ^***^ *p* < 0.001.

Using western blot analysis, we further quantified the levels of EMT markers expressed in cells overexpressing MFAP5. As shown in Figure 4, MFAP5 overexpression resulted in an increase in E-cadherin and a decrease in N-cadherin and α-SMA. These findings suggest that EMT in EC cells may be inhibited by overexpressing MFAP5.

### 3.5 MFAP5 overexpression inactivates the AKT/mTOR pathway

**Figure 5:**
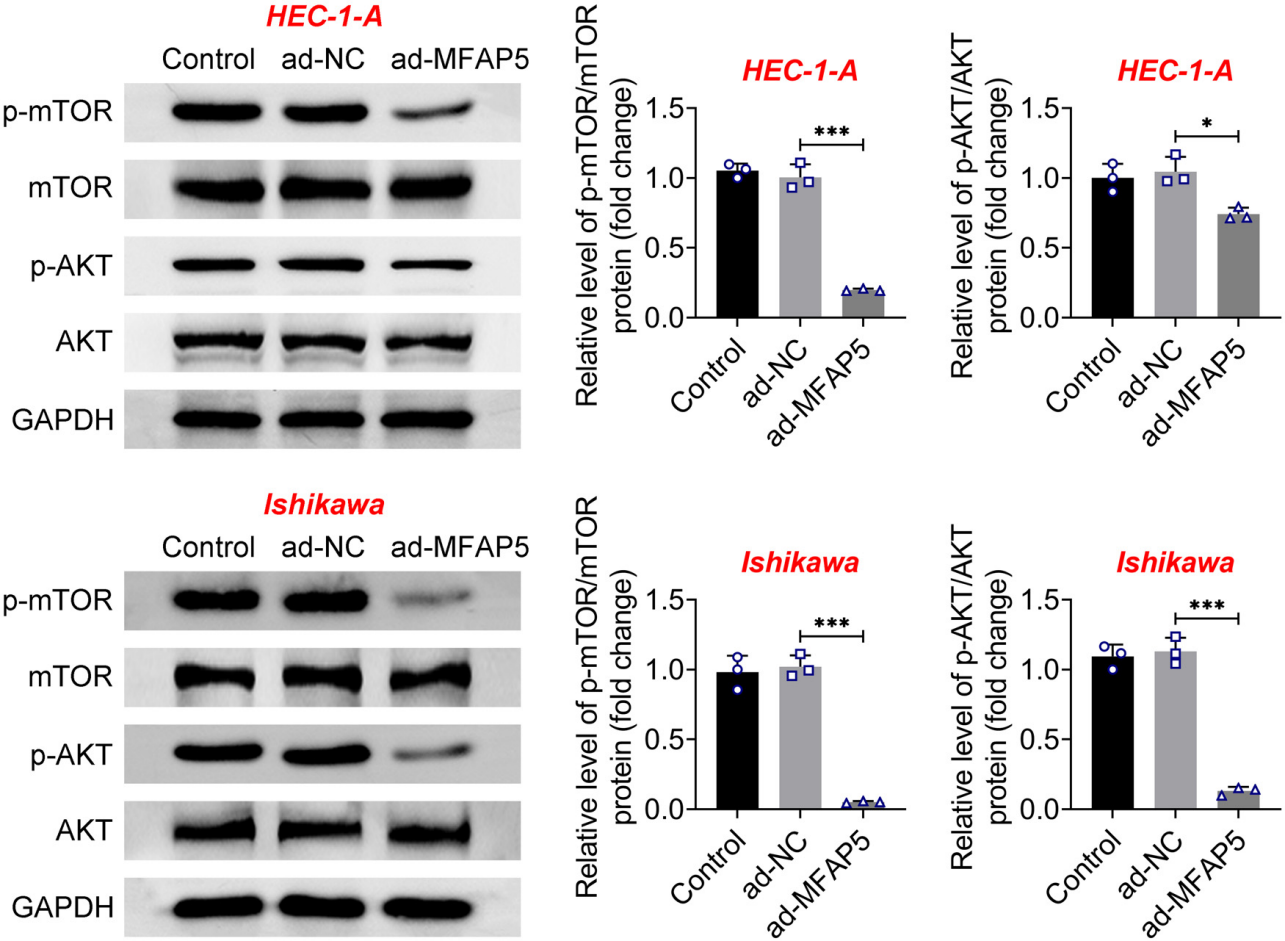
MFAP5 overexpression inactivates the AKT/mTOR pathway. Protein expressions of AKT, p-AKT, mTOR, and p-mTOR were determined by western blot assays. ^*^ *p* < 0.05 and ^***^ *p* < 0.001.

Next, the phosphorylation of AKT and mTOR was monitored to explore whether overexpressing MFAP5 in EC cells mediated the activity of the AKT/mTOR pathway. Western blot analysis revealed that phosphorylations of AKT and mTOR were reduced when MFAP5 was overexpressed in EC cells (Figure 5). These data suggest that MFAP5 overexpression decreased AKT/mTOR activity.

## 4 Discussion

Poor prognosis of EC is largely determined by tumor metastasis [14,15]. Patients with cancer cells spreading to other organs make treatment more challenging, and the prognosis worsens [16,17]. Therefore, investigating the mechanisms of tumor metastasis in EC may help to develop effective treatment strategies to improve patient outcomes. In this study, reduced level of MFAP5 was tested in EC cells, and cell proliferation was suppressed while cell apoptosis was promoted by overexpressing MFAP5. Importantly, our results demonstrate that MFAP5 overexpression inhibited migration, invasion, and EMT of EC cells. These data show a vital role of MFAP5 in EC cell malignant behaviors.

MFAP5, a matrix glycoprotein consisting of microfibers, serves as a multifunctional protein implicated in endothelial cell behavior and mediated cell adhesion [18,19]. Recently, most research studies indicated that MFAP5 expression is high in various tumor types, leading to enhanced tumor proliferation, endothelial cell motility, and angiogenesis [11–13,20,21]. For example, MFAP5 has been reported to enhance the stem cell features of non-small cell lung cancer cells, while the knockdown of MFAP5 has been shown to exert the opposite effect [22]. Another study has found that MFAP5 silencing resulted in apoptosis and growth inhibition in cervical cancer cells [12]. Herein, our TCGA database analysis revealed contrasting findings, showing underexpression of MFAP5 in EC tumor tissues, which differs from previous studies. Later, we detected diminished MFAP5 expression in EC cells. The invasion and metastasis of tumor cells are intricately regulated by the ECM, with MMPs, particularly MMP2 and MMP9, playing a crucial role in facilitating tumor formation and metastasis by degrading matrix proteins [23]. Dysregulation of MMPs can compromise the integrity of tissue barriers, facilitating tumor invasion [24]. This process promotes the degradation of the ECM and basement membrane, thereby enhancing tumor penetration, invasion of adjacent tissues, and metastasis to distant sites [25]. Our findings demonstrate that MFAP5 overexpression led to a decrease in MMP2 and MMP9 levels, thereby confirming the inhibitory effect of MFAP5 on EC cell migration and invasion. However, how MFAP5 affects MMPs expression should be investigated further. EMT is a biological phenomenon in which cancer cells undergo a phenotypic change from epithelial to mesenchymal cells, resulting in enhanced abilities for proliferation, migration, and resistance to apoptosis [26–28]. MFAP5 has been shown to play a role in EMT process in cancer progression [11,21,29]. Similar to previous results, our further data found an increased level of E-cadherin and a decreased N-cadherin and α-SMA in cells overexpressing MFAP5, indicating that MFAP5 overexpression inhibited EMT in EC cells.

AKT, a protein kinase, can be activated by various growth factors, subsequently activating mTOR, thus promoting cellular proliferation and growth [30,31]. In addition, EMT can be accelerated by activating AKT [32,33]. Research indicates that dysregulated activation of the AKT/mTOR pathway in EC tissues can enhance cancer proliferation, invasion, and metastasis, while suppressing apoptosis and accelerating tumor malignancy [34–36]. Consequently, the modulation of the AKT/mTOR pathway has emerged as a prominent focus in the realm of EC therapy. In this study, the activity of the AKT/mTOR pathway was found to be reduced in EC cells overexpressing MFAP5, manifested by the inhibition of AKT and mTOR phosphorylation levels. However, further experiments will be required to determine whether MFAP5 exerts its biological role in EC by mediating the AKT/mTOR pathway. In addition, whether MFAP5 has the same biological function *in vivo* and the further regulatory mechanisms need in-depth investigation.

**Figure 6:**
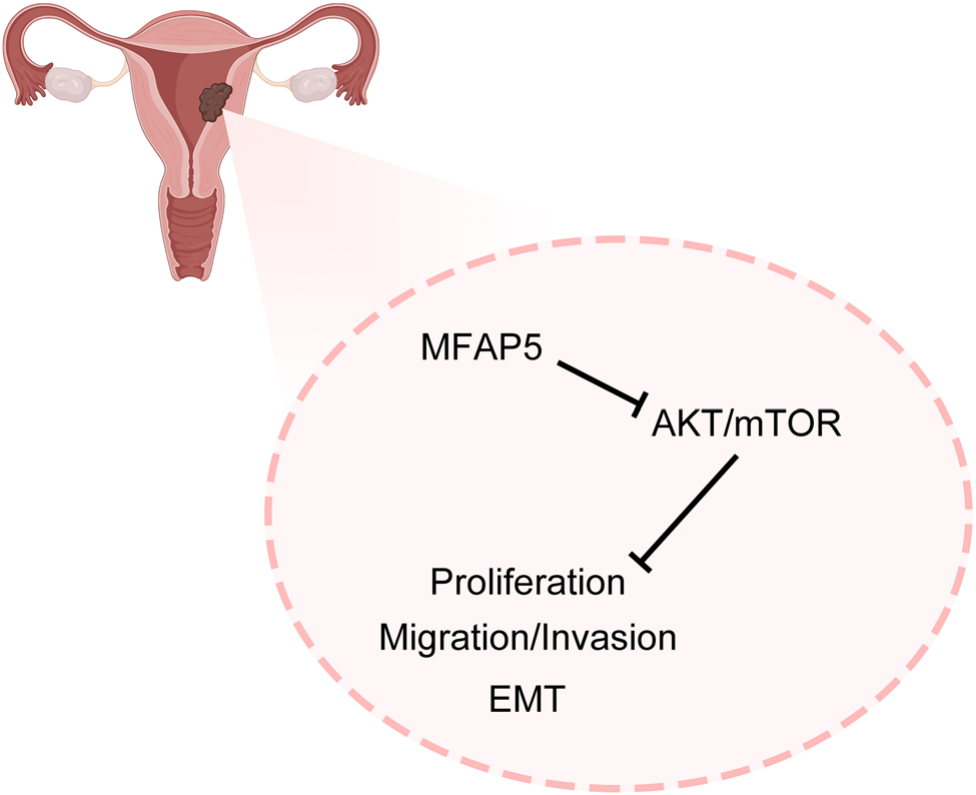
Schematic diagram of MFAP5 in EC via AKT/mTOR pathway. MFAP5 overexpression inhibited the activation of the AKT/mTOR pathway, thereby suppressing the proliferation, migration, invasion, and EMT of EC cells.

In conclusion, our findings confirmed that MFAP5 regulates the EMT and malignancy in EC cells, and inactivation of the AKT/mTOR pathway may be one of the mechanisms (Figure 6), suggesting that targeting MFAP5 may represent an attractive strategy for EC treatment.
